# *Mimiviridae*: An Expanding Family of Highly Diverse Large dsDNA Viruses Infecting a Wide Phylogenetic Range of Aquatic Eukaryotes

**DOI:** 10.3390/v10090506

**Published:** 2018-09-18

**Authors:** Jean-Michel Claverie, Chantal Abergel

**Affiliations:** Aix Marseille Univ, CNRS, IGS, Structural and Genomic Information Laboratory (UMR7256), Mediterranean Institute of Microbiology (FR3479), 163 Avenue de Luminy, F-13288 Marseille, France; chantal.abergel@igs.cnrs-mrs.fr

**Keywords:** *Mimiviridae*, algal virus, giant virus, *Phycodnaviridae*, aquatic virus

## Abstract

Since 1998, when Jim van Etten’s team initiated its characterization, *Paramecium bursaria* Chlorella virus 1 (PBCV-1) had been the largest known DNA virus, both in terms of particle size and genome complexity. In 2003, the *Acanthamoeba*-infecting Mimivirus unexpectedly superseded PBCV-1, opening the era of giant viruses, i.e., with virions large enough to be visible by light microscopy and genomes encoding more proteins than many bacteria. During the following 15 years, the isolation of many Mimivirus relatives has made *Mimiviridae* one of the largest and most diverse families of eukaryotic viruses, most of which have been isolated from aquatic environments. Metagenomic studies of various ecosystems (including soils) suggest that many more remain to be isolated. As *Mimiviridae* members are found to infect an increasing range of phytoplankton species, their taxonomic position compared to the traditional *Phycodnaviridae* (i.e., etymologically “algal viruses”) became a source of confusion in the literature. Following a quick historical review of the key discoveries that established the *Mimiviridae* family, we describe its current taxonomic structure and propose a set of operational criteria to help in the classification of future isolates.

## 1. Introduction

The viral nature of Mimivirus was finally recognized in 2003 [1,2], more than 10 years after Timothy Rowbotham, an investigator for Public Health England, first isolated it from a cooling tower in the city of Bradford when searching for a pneumonia-causing pathogen [3]. For 10 years, the newly isolated *Acanthamoeba*-infecting microbe, dubbed Bradfordcoccus, was unsuccessfully investigated as an intracellular parasitic bacterium, resisting all standard characterization approaches. The difficulties, however, were not technical but epistemological [4,5]. After more than a century of virology dedicated to the study of “filtering” infectious agents originally defined as those not retained by sterilizing filters and invisible by light microscopy, the notion that a virus could be propagated by particles as big as bacteria was a conceptual leap that no *bona fide* virologist was ready to make. It was thus for a team of bacteriologists to identify Mimivirus as the first “giant” virus, which then allowed the subsequent discovery of many more of them that now encompass at least four different families and have opened a new and booming era in virology [4].

The initial awe caused by the mere size of the giant virus particles was further amplified by the discovery that they packed giant genomes encoding more genes than many bacteria and the smallest parasitic eukaryotic microorganisms [4,6,7]. This revived numerous reflections and debates on the concept of virus [4,6,8,9,10,11,12,13,14,15], on their position relative to the Tree of Life [16,17,18,19,20,21,22,23,24,25], and on their role in the evolution of cellular life forms [4,10,14,26,27,28]. The giant virus revolution is clearly not near its end, and more surprises are to come. One of them is the continuing and even accelerating discovery of Mimivirus relatives in fresh, brackish, and seawater environments, already making *Mimiviridae* a prominent family among aquatic viruses (Table 1). Members of this rapidly increasing family are both remarkable by the phylogenetic diversity of their hosts and the unmatched variability of their particle and genome sizes. Following a short history of early developments, we then review the most recent discoveries of aquatic *Mimiviridae*, before ending with a set of guidelines to help their correct classification amidst other established families of aquatic viruses.

## 2. Results

The saga of giant viruses truly began with the pioneering work of Jim van Etten and his collaborators who discovered, isolated, and extensively characterized the first large DNA viruses infecting a protist. This protist was the unicellular freshwater green algae *Chlorella* (*Trebouxiophyceae*, *Viridiplantae*), with small round cells that are 3 µm in diameter and are nowadays quite popular as a detoxifying nutritional supplement in health food stores. Ironically, *Chlorella* are linked with the history of virology from the very beginning, since they were discovered by the same famous Dutch microbiologist Martinus W. Beijerinck, who coined the term “virus” (even though its concept of “liquid” infectious agent was quite wrong) [29]. Thus, with a little twist of fate, Beijerinck could have discovered *Chlorella*-infecting viruses instead of simply reproducing Ivanovsky’s work on the filterable tobacco mosaïc virus [30]. No doubt that the history of virology would then have been entirely different [5] and the discovery of giant viruses might not have been delayed until the 21st century.

### 2.1. Pionnering Work on Large Aquatic Viruses

The first descriptions of very large icosahedral viruses infecting *Chlorella* were published in 1981 [31,32], which was very soon after Torrella and Morita first reported the large incidence of virus-like particles in coastal seawater in their landmark 1979 article [33]. An interesting twist (most often forgotten) of their landmark work is that they observed populations of unusually large viruses, collected on a 0.2-µm pore size filter, meant to retain bacteria. Unfortunately, the whole field of marine virology was subsequently based on the analysis of “viral fractions”, defined as the filtrates in such filtration protocols [34], thus coming back to the historical definition of viruses as “filterable” microbes. This again probably delayed the discovery of giant marine viruses by decades.

Once Van Etten and collaborators realized that the easiest virus to work with was one infecting a symbiotic *Chlorella* of paramecium (the now famous *Paramecium bursaria* Chlorella virus 1, PBCV-1), its characterization proceeded quickly. Much before the completion of its genome sequencing in 1997 [35], they correctly estimated as soon as 1982 that its 190-nm-diameter icosahedral particle contained a dsDNA genome more than 300 kb long [36]. The stage was then already set for the new notion of giant viruses defined in reference to both their large particle sizes and large genomes. Variable thresholds are used in today’s literature but are around 350 nm for particle size (i.e., making them visible by light microscopy) and 300 kb for genome length [4]. Historically, the term “giant viruses” was first used in 1995 by Jim Van Etten in the title of an article published in a Korean journal [37], then again in 1999 [38]. Following the subsequent threshold values introduced by the revolution of the truly giant viruses initiated by Mimivirus [1,2,3,4], Jim Van Etten nowadays qualifies the *Chlorella* viruses as “very large” instead of giant. As we progress in our knowledge about their structure and metabolic capacity, it becomes clear that no fundamental biological difference distinguishes very large from giant dsDNA viruses that even coexist in the same *Mimiviridae* family.

### 2.2. From Acanthamoeba Polyphaga Mimivirus (APMV) to the First Marine Mimiviridae

Our determination of the complete genome sequence of Mimivirus in 2004 [2] happened to coincide with a number of unrelated developments in the field of aquatic/marine microbiology that led to us to change the course of our own research. One was the seminal publication of the first environmental “shotgun” sequencing of the Sargasso Sea by Venter’s team [39]. The second one was the fourth Algal Virus Workshop (4th AVW) [40], in which we participated at the invitation of Jim Van Etten, whom we had contacted as the leading expert in very large viruses. In collaboration with Dr. E. Ghedin (then, from the TIGR Institute), we screened Venter’s first marine metagenomics sequence database with the freshly determined Mimivirus sequence. To our surprise, 15% of predicted Mimivirus ORFs had their closest homologs in these marine environmental sequences [41]. A few months later, our attendance at the 4th AVW (and exchanges with its amazingly congenial research community) made us aware that unicellular algae were indeed hosts of many large viruses. Following these two hints, we first confirmed in silico the presence of Mimivirus relatives in the sea using metagenomic datasets [42], gene-targeted amplicons sequences [43], and the partial genome sequences of previously isolated viruses infecting phylogenetically distant unicellular marine algae [44]. We finally succeeded in isolating the closest marine Mimivirus relative, *Megavirus chilensis*, from water sampled off the coast of central Chile [45]. In contrast with the other known marine *Mimiviridae* members, *M. chilensis* infects *Acanthamoeba*, i.e., the original laboratory host of Mimivirus. With its 1.26-Mb genome predicted to encode 1120 proteins, *M. chilensis* remained the largest *Acanthamoeba*-infecting *Mimiviridae* until the recent isolation and characterization of two new aquatic isolates called Tupanviruses [46] (see below) (Table 1).

### 2.3. C. Roenbergensis Virus: The Prototype of a New Subfamily within the Mimiviridae

**Figure 1:**
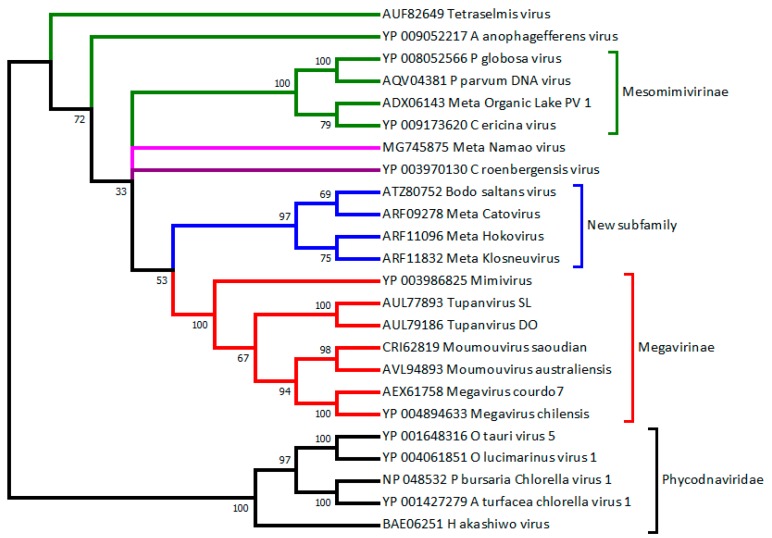
Phylogeny of *Mimiviridae* vs. *Phycodnaviridae* based on DNA polymerase B. Diverse algae-infecting “Phycodnaviruses” (prasinovirus OtV5, OlV1; raphidovirus HaV1; chlorovirus PbCV1, AtCV1) clearly cluster as an outgroup (with high confidence). The *Mimiviridae* members cluster in different proposed subfamilies (from the bottom up): The “*Megavirinae*” (in red, closest to Mimivirus), a new clade including BsV (in blue, mostly defined by Klosneuvirus-like metagenomics assemblies), the *Mesomimivirinae* (in green, with PgV and CeV). These proposed subfamilies are highly supported (bootstrap values > 97%). In contrast, CroV (in indigo), Namao virus (in purple), AaV, and TetV (in green) remain isolated. This tree was produced from an alignment of 24 sequences (519 sites) using neighbor joining and the JTT substitution model. Bootstrap values are indicated on each branch. These computations were performed on the MAFFT online service [50]. NCBI accession numbers are given for each sequence. The prefix “Meta” indicates sequences from nonisolated viruses. A more generic phylogenetic tree placing the *Mimiviridae* in the context of other large DNA virus families is shown in [51].

**Figure 2:**
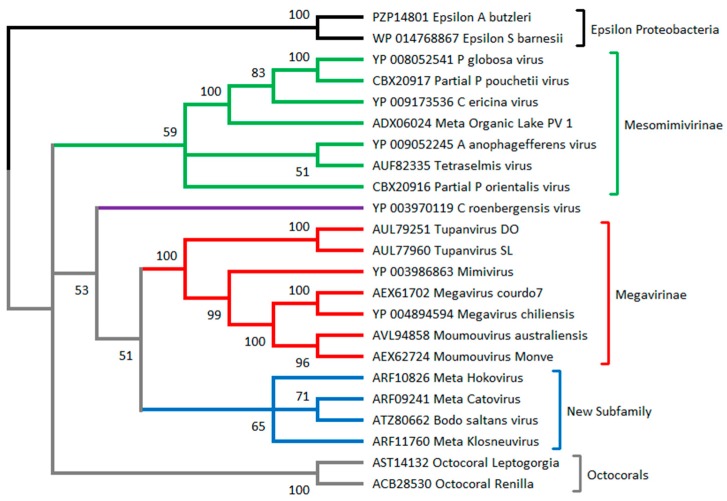
Phylogeny of *Mimiviridae* based on MutS7. MutS sequences from Epsilon proteobacteria (from the genera *Arcobacter* and *Sulfurospirillum*) are used as the root, as they are most likely the source of the *Mimiviridae* MutS7 gene (MutS homologs are present throughout the epsilon division). The tree suggests with great confidence that the mitochondrial MutS present in octocorals (in grey) and that are present in all *Mimiviridae* have a common origin. The clustering of the *Mimiviridae* into several subfamilies is mostly consistent with that suggested by the DNA polymerase phylogeny (Figure 1). The algae-infecting *Mimiviridae* (in green) now appears monophyletic. This tree was produced from an alignment of 23 sequences (479 sites) using neighbor joining and the JTT substitution model. Bootstrap values are indicated on each branch. The computations were performed on the MAFFT online service [50]. NCBI accession numbers are given for each sequence. The prefix “Meta” indicates sequences from nonisolated viruses. The prefix “Partial” indicates sequences from amplicons.

A large virus was isolated from the coastal waters of Texas in the early 1990s. Its host, originally misidentified as *Bodo* sp., was *Cafeteria roenbergensis* (CroV), a 2–6-μm-long bicosoecid heterokont phagotrophic flagellate (Stramenopiles) that is widespread in marine environments [47]. In 2010, the complete genome sequence of *C. roenbergensis* virus suggested the existence of a distinct clade within the *Mimiviridae* [48] (Figure 1 and Figure 2). It was also the first demonstration that Mimivirus relatives could infect very distant hosts (*Acanthamoeba* and *C. roenbergensis*), belonging to the earliest diverging branches within the Eukaryota, the unikonts, and the bikonts [49]. As it is devoid of the thick fiber layer surrounding the icosahedral capsids characteristic of the *Acanthamoeba*-infecting *Mimiviridae*, CroV’s particle is only 300 nm in diameter. Yet, its 693-kb genome (predicted to encode 544 proteins) remained the largest of all marine plankton-infecting viruses until recently (see Section 2.5). As a new, distinct member of the family, CroV played an important role in delineating both the conserved features and the expected diversity of the *Mimiviridae* in terms of gene content, particle size, morphology, and replication process. These conserved features can be used as criteria to recognize and classify future family members, despite the further diversity exhibited by emerging clades. These features are presented in Section 3.2.

### 2.4. Smaller Mimiviridae Infecting Bona Fide Microalgae: Yet Another Subfamily

Prior to the complete genomic characterization of Mimivirus, a number of viruses infecting various species of unicellular algae had been isolated. These viruses exhibiting large dsDNA genomes packed in large icosahedral particles were propagated on cultures of *Pyramimonas orientalis* (*Chlorophyta*, *Prasinophyceae*), *Phaeocystis pouchetii* and *P.globosa* (*Haptophyta*, *Prymnesiophyceae*), and *Chrysochromulina ericina* (*Haptophyta*, *Prymnesiophyceae*) [52,53,54]. Targeted amplicons of genes encoding DNA polymerase B [43] or capsid proteins [55] produced partial sequences, some of which suggested a phylogenetic affinity between the largest algal viruses and Mimivirus. However, given the possibility of lateral gene transfers, complete genomes sequences were required to confirm the existence of a distinct clade of algae-infecting *Mimiviridae*. Such a clade was clearly confirmed following the analyses of the complete genome sequences of *P. globosa* virus (PgV) in 2013 [56], of *Aureococcus anophagefferens* virus in 2014 [57], and *C. ericina* (now *Haptolina ericina*) virus (CeV) in 2015 [58] (Table 1, Figure 1 and Figure 2). The host spectrum of phytoplanckton-infecting *Mimiviridae* was recently extended to *bona fide* green algae (e.g., members of the *Chlorophyta* lineage) with the detailed characterization of a virus (TetV) infecting Tetraselmis [59]. TetV exhibits the largest genome (668 kb) sequenced to date for a virus that infects a photosynthetic organism (Table 1). Yet, the diameter of its icosahedral virion (≈240 nm) remains smaller than virus-like particles (up to 500 nm) observed long ago in other green algae [60,61]. This suggests that more findings are expected as new algal hosts will be investigated. As of today, the known host spectrum of algae-infecting *Mimiviridae* (green branches in Figure 1 and Figure 2) thus encompasses several classes of the *Heterokonta* superphylum: *Haptophyceae* (PgV, CeV), *Pelagophyceae* (AaV), and *Chlorodendrophyceae* (TetV)).

### 2.5. Recent Isolations of New Truly Giant Mimiviridae Members 

As we started to think that the upper limits of *Mimiviridae* genome size and gene content had been reached with the 1,259,197 bp and 1120 predicted proteins of *M. chilensis*, the year 2018 brought in three record-breaking viruses. Two of them, called Tupanvirus soda lake (TupanSL) (isolated from an alkaline lake in Brazil) and Tupanvirus deep ocean (TupanDO) (isolated from 3000 m deep marine sediments), can be propagated in the laboratory on *A. castellanii* and *Vermamoeba vermiformis* [46]. Their linear genomes consist of 1,439,508 bp and 1,516,267 bp, predicted to encode 1276 and 1425 proteins, respectively. Not far behind but infecting a very different host, Deeg et al. [62] isolated the kinetoplastid-infecting *Bodo saltans* virus (BsV), exhibiting a 1.39-Mb genome predicted to encode 1227 proteins. Interestingly, both types of viruses hint at the existence of further subdivisions in the growing *Mimiviridae* family (Figure 1 and Figure 2).

### 2.6. Metagenomic Contributions to the Expansion of the Mimiviridae Family

We strongly believe that the description and taxonomic analysis of a brand new virus family, such as *Mimiviridae*, should imperatively rely on laboratory cultivable isolates, the availability of which is required for a detailed description of the infectious cycle, which should be similar throughout the family. The exchange of virus isolates between laboratories should remain a key requirement for independent scientific validation and is obviously needed for collaboration purposes. It should also be emphasized that most viral sequences produced from environmental DNA are so alien that they can only be detected and interpreted by comparison with sequences determined from previously isolated viruses. This being said, the complete history of *Mimiviridae* could not be told without mentioning some significant contributions from metagenomic studies.

We previously cited the contribution of the early Sargasso Sea data to establish the presence of Mimivirus relatives in the sea [41]. This was further confirmed in the larger Global Ocean Sampling dataset [63] and was associated with algal viruses through their partially sequenced DNA polymerases [43].

Once a special version of the DNA mismatch repair protein (MutS7)—curiously shared with the octocoral mitochondria [64]—was recognized as a distinctive feature of *Mimiviridae*, it also served to estimate their abundance in the marine environment [44].

The metagenomics analysis of a hypersaline lake in the Antarctic by Yau et al. [65] inaugurated a new era by claiming the discovery of two new *Mimiviridae* members based on two large contigs assembled from environmental sequences. Their claim was further supported by the full genome assembly of a virophage, then known as a unique parasite of Mimivirus [66,67], from the same data. This article was quite influential in prompting the search for additional virophages in a large variety of environments [68,69,70,71]. However, it was also a source of taxonomic confusion, as the authors named their two new large viruses “Organic Lake Phycodnavirus 1 and 2” (abbreviated as OLPV-1, OLPV-2), based on their phylogenetic affinity with viruses (CeV and PgV infecting the unicellular algae *Chrysochromulina* and *Phaeocystis*. Like other algae-infecting Mimivirus relatives, these viruses are now believed to define a *Mimiviridae* subfamily (see Section 2.4) (Figure 1 and Figure 2), although they often remain clumsily referred to as “OLPV-like” in the literature.

The more recent worldwide *TARA* OCEANS expeditions [72,73] confirmed the abundance of marine *Mimiviridae* (also called *Megaviridae*) in the ocean, although most of the sequences classified in this family showed a large divergence from known viral genomes. This suggests that we need more isolates to adequately span the diversity of the family (as confirmed by the most recent findings, see Section 2.5). The specific search for viral contigs exhibiting genes coding for the glutamine-dependent asparagine synthase uniquely found in *Mimiviridae* also hinted at the existence of additional clades, for which isolated representatives are lacking [74].

The need for such missing isolates is well illustrated by a recent study performed by Schulz et al. [75]. Following a metagenomics analysis of a wastewater treatment plant in Klosterneuburg, Austria, these authors identified a divergent Mimivirus-relative (called “Klosneuvirus”) with an estimated genome size of 1.57 Mb, dwarfing the size of all previously isolated and fully sequenced *Mimiviridae*. In the absence of a physical isolate, such a genome size remains to be confirmed, as it is obtained by summing 22 separate contigs (none of them larger than 452 kb) predicted to be part of the same genome by a heuristic bioinformatics procedure called “binning”. A similar uncertainty is shared by three other predicted Klosneuvirus relatives (i.e., Catovirus, Hokovirus, Indivirus). Despite the lack of physical isolates, the authors have proposed to classify them in a new subfamily (the “Klosneuvirinae”) within the family *Mimiviridae*. Fortunately, the isolation of BsV (Section 2.5) one year later appears to substantiate this new clade (Figure 1 and Figure 2). However, despite its phylogenetic similarity, BsV is conspicuously lacking the key feature of the postulated Klosneuvirinae: their large sets of amino-acyl tRNA synthetases (up to 19 for Klosneuvirus, only 2 for BSV).

The latest unexpected addition to the family *Mimiviridae* might be a virus that can cause a lethal disease in the lake sturgeon *Acipenser fulvescens* [76], called Namao virus. The genomic data presumably associated to this virus consists of two non-overlapping contigs totaling 306,448 bp, sequenced from DNA extracted from a homogenate of 3 tissues of 16 infected fishes [77]. There is yet no proof that these two contigs belong to the same virus. The remote possibility also remains that these sequences might correspond to ancestral viral sequences integrated in the fish genome, as previously seen in other organisms [78,79]. Once definitely established by the full characterization of a cloned Namao virus isolate, the confirmation that some *Mimiviridae* members could include vertebrate species would make the host range of this family (from algae to fish!) truly unmatched among known (aquatic) viruses.

## 3. Discussion

### 3.1. Algae-Infecting Mimiviridae versus Phycodnavirididae

The discovery of an increasing number of Mimivirus relatives infecting algae has generated some confusion in the literature about their taxonomic assignment. This confusion is propagated through the nonofficial classification assigned to their associated genomic entries by leading reference databases such as NCBI [80]. Completely sequenced algae-infecting *Mimiviridae* can be found erratically classified as members of the *Phycodnaviridae* family in the genus *Prymnesiovirus* (PgV) as “unclassified *Phycodnaviridae*” (AaV, CeV) or as “unclassified virus” (TetV-1). Among partially sequenced *Mimiviridae* candidates (inferred from metagenomics assemblies), the naming and classification of two “Organic Lake Phycodnaviruses” (OLPV-1 and OLPV-2) has been particularly misleading. Others include *Phaeocystis pouchetii* virus (PpV-01) and *Pyramimonas orientalis* virus (PoV-01B). These erroneous classifications are not detected by nonspecialists and do affect the outcome of automated large-scale phylogenetic analyses and gene content comparisons, such as the delineation of core gene sets.

The difficulties in classifying new algae-infecting large DNA viruses are also inherent in the historical definition of the *Phycodnaviridae* family. According to its official ICTV definition, the family is meant to include algae-infecting viruses with large icosahedral virion (Ø > 100 nm) and large genome (>100 kb). The family was then further divided into five genera based on the algal host (Table 2). We believe there are some fundamental flaws in this historical design. First, as algae are among the most phylogenetically diverse organisms (both by the origin of their chloroplast and that of the eukaryotic recipient), we could not expect that a single viral family (nowadays suggesting a monophyletic group for most authors) would adequately represent the diversity of their viruses. Indeed, the phycodnaviruses from the various genera appear increasingly different as we learn more about their infection process, their replication cycle, their genome structure, and their gene content. No other established viral family would encompass viruses with enveloped versus nonenveloped virions, with different genome structures (circular, linear), or encoding or not encoding a transcriptional apparatus. In our opinion, today’s genera composing the *Phycodnaviridae* should become separate families.

The current genus partition, designed according to the taxonomy of the algal host, is similarly unsatisfactory, as it suggests a one-to-one correspondence between virus and host type, which we know is obviously wrong (e.g., viruses from many different families infect human). The diversity of the viruses infecting the *Prymnesiophyceae* (*Haptophyceae*) is a good example of this problem. Two types of very different dsDNA Prymnesioviruses have been shown to infect the unicellular algae *Phaeocystis* [54]. This is also the case of Tetraselmis, which can be infected by a smaller virus (60-nm diameter, 31-kb genome) [81] (in addition to RNA viruses, not discussed here). It is now clear that the largest virion/genome type (highlighted in orange in Table 2) belongs to the *Mimiviridae* [56], as well as other *Haptophyceae*-infecting viruses and the so-called Organic Lake “phycodnaviruses” [58]. Moreover, the *Phycodnaviridae* family includes a “*Coccolithovirus*” genus corresponding to a third type of virus infecting *Emiliania huxleyi* that is also a *Prymnesiophyceae*. Thus, even within a single host genus, the current partition does not correctly reflect the diversity of the “*Phycodnaviridae*”. More problems can be anticipated with the green dinoflagellate *Heterocapsa circularisquama* (*Dinophyceae*), infected by a large DNA virus which might be close to the *Asfarviridae* [82] originally described as causing swine fever in Africa.

### 3.2. How to Recognize and Classify Future Members of the Mimiviridae

Our opinion is that a virus family should only include viruses sharing a number of key properties likely to have been inherited from a common “founding” ancestor. These common properties will in turn imply the presence and conservation of orthologous proteins, each of which should have its most similar homolog within the family (with rare exceptions caused by horizontal transfers). These key properties/proteins will be linked to a similar structure of the virion (e.g., capsid proteins), the genome replication machinery (DNA replication and repair enzymes), the virus cycle dependency of nuclear functions (e.g., the transcription machinery), or the intracellular location (e.g., nuclear/cytoplasmic) of particle production. Proteins/enzymes uniquely present in a group of viruses will be particularly useful as family “signature” even though they will be useless in the context of broader phylogenetic analyses. Finally, we do expect that the physiological traits shared by these viruses will make their global gene contents qualitatively similar. Members of the same virus family should then appear well clustered in a cladistic analysis/tree [51,62].

The number of available *Mimiviridae* complete genome sequences is now large enough to make each new member easily recognizable by global protein level similarity searches. Even though the four most recent *Mimiviridae* isolates (TetV, BsV, Tupanviruses) exhibit a variable fraction of predicted protein best matching viral database entries, more than 60% are from previously characterized members of the family.

Confirmatory evidence is readily provided by the positioning of *Mimiviridae* candidates in the phylogenetic trees built from sequence alignments or key enzymes such as B-type DNA polymerase, virion packaging ATPase, major capsid protein (AaV, TetV, CeV), and three types of proteins (namely, NCVOG0038, NCVOG0022, NCVOG0249) with homologs in other families of large dsDNA viruses [84].

The previous genomic characterizations of *Mimiviridae* members led to the discovery of various key features apparently unique to the family. The association of any of them with a candidate virus (even partially sequenced) will considerably strengthen its tentative classification as a new member of the *Mimiviridae*. These key findings include:

1. A high prevalence of the strictly conserved *AAAATTGA* motif in the promoter regions of early transcribed genes [85].
2. A high prevalence of hairpin-forming transcription termination motifs in the 3′ end of genes [86].
3. The co-isolation (or sequencing) of a virophage. Virophages are small dsDNA viruses with 17–18-kb genomes that can only replicate in host cells undergoing an infection by *Mimiviridae* [67,87]. Not all family members have been associated with a virophage (Table 3).
4. The detection of a transpoviron. Transpovirons are 7-kb-long plasmid-like linear dsDNA molecules found in association with Mimivirus-relatives [88].
5. The presence of a gene encoding a *Mimiviridae*-specific version of glutamine-dependent Asparagine synthetase (AsnS). A different, easily distinguishable homolog of this enzyme is encoded by some Prasinoviruses (e.g., OtV5) [74].
6. The presence of amino-acyl tRNA synthetases (aaRS). The finding of such central components of the translational apparatus in the Mimivirus genome [2] was considered revolutionary, as the historical definition of viruses denied them the capacity to synthetize proteins [4]. Conflicting hypotheses on the evolutionary origin of these viral enzymes generated a still ongoing controversial debate. The presence of these aaRS remains a remarkable feature of the *Mimiviridae*, although not unique to them anymore [7]. Unfortunately, the number of virus-encoded aaRS varies greatly among the *Mimiviridae* members (from 0 up to 20 different ones in Tupanviruses [46]) (Table 3), which reduces the utility of their detection for classification purpose. However, the presence of aaRS in a virus genome remains a strong complementary argument for their classification within the *Mimiviridae*.

The statistical nature of features 1 and 2 make them less decisive than features 3–6, although none of the latter are present in all members (Table 3).

As of today, the sole protein that is unique to the *Mimiviridae* and is found in all fully sequenced genomes is a special version of a homolog to a DNA mismatch repair enzyme (MutS7). MutS proteins are ubiquitous in cellular organisms. However, the *Mimiviridae* MutS7 version is curiously related to the one found in ɛ-proteobacteria and in the mitochondrial genomes of octocorals (Figure 2) [12,44]. Detecting MutS7 homologs in DNA sequences of newly isolated viruses is thus a strong argument in favor of their classification within the *Mimiviridae*. Thanks to their strong phylogenetic signals, both AsnS and MutS7 can be used as “baits” to detect presumed *Mimiviridae* members in large metagenomic sequence datasets [44,74].

## 4. Conclusions

Fifteen years after the publication of its prototype Mimivirus, the *Mimiviridae* now appears as a major family of aquatic viruses [89]. Retrospectively, it is quite fortunate that the name “Mimivirus” (for microbe-mimicking virus) was forged without reference to the original *Acanthamoeba* host. Viruses from the *Mimiviridae* family are now known to infect the most phylogenetically diverse spectrum of eukaryotic unicellular organisms, ranging across six major phyla: *Amoebozoa* (Mimivirus), *Haptophyta* (PgV), *Chlorophyta* (TetV), *Excavata* (BsV), *Heterokonta* (CroV), and perhaps *Opisthokonta* (Sturgeon virus). In addition to its host diversity, the *Mimiviridae* family is also the one exhibiting the broadest distribution of genome sizes (from 370 kb for AaV to 1.51 Mb for TupanDO) as well as of particle sizes (from 750 to 140 nm for icosahedral virions, up to 2.3 µm for the tailed Tupanviruses). Despite these huge differences, all currently known members of the family appear to propagate in particles with similar architecture (including an internal lipid membrane) and replicate in the host cytoplasm using their own well-conserved transcription machinery. Yet, their gene contents are also extremely variable, the intersection of which only amounts to 30 strictly conserved core genes [58]. Future work may thus lead to a partition of the current *Mimiviridae* family into smaller more homogenous distinct families, as we suggested for the *Phycodnaviridae*.

## Figures and Tables

**Table 1:** Physically isolated *Mimiviridae* with fully sequenced genomes.

Name ^1^	Accession	Genome Size (kb)	Virion Type	Virion Size (nm)	Host Phylum
Mimivirus ^2^	NC_014649	1181	icosahedron	750	Amoebozoa
Megavirus ^2^	NC_016072	1259	icosahedron	680	Amoebozoa
Moumouvirus ^2^	NC_020104	1021	icosahedron	620	Amoebozoa
CroV	NC_014637	693	icosahedron	300	Heterokonta
PgV	NC_021312	460	icosahedron	150	Haptophyceae
CeV	NC_028094	474	icosahedron	160	Haptophyceae
TetV	KY322437	668	icosahedron	240	Chlorophyta
BsV	MF782455	1386	icosahedron	300	Excavata
AaV	NC_024697	371	icosahedron	140	Heterokonta
TupanSL	KY523104	1439	icosa. + tail	450 + 550	Amoebozoa
TupanDO	MF405918	1516	icosa. + tail	450 + 550	Amoebozoa

^1^ Abbreviations: CroV: *Cafeteria roenbergensis* virus; PgV: *Phaeocystis globosa* virus; CeV: *Chrysochromulina ericina* virus; TetV: Tetraselmis virus; BsV: *Bodo saltans* virus; AaV: *Aureococcus anophagefferens* virus; TupanSL: Tupanvirus soda Lake; TupanDO: Tupanvirus deep Ocean. ^2^ Several strains very similar to these prototypes have been isolated and fully sequenced. All listed viruses have been isolated from aquatic environments.

**Table 2:** Structure of the *Phycodnaviridae* family as presently recognized by ICTV.

Genus	Prototype	Encoded RNA pol	Genome Size (kb)	(G + C) %	Accession	Virion Ø (nm)
Chlorovirus	PBCV-1	No	330	40	NC_000852	190
Coccolithovirus ^1^	EhV-86	Yes	407	40.2	NC_007346	180
Phaeovirus	EsV-1	No	336	51.7	NC_002687	200
Prasinovirus	OtV-5	No	187	45	NC_010191	120
Prymnesiovirus I ^2^	PgV-16T	Yes	460	32	NC_021312	153
Prymnesiovirus II ^2^	PgV-01T	Unknown	≈177	Unknown	-	106
Raphidovirus	HaV-1	No	275	30.4	KX008963	202

^1^ Enveloped capsid [83]; ^2^ Two distinct virus groups [54]. Prymnesiovirus I corresponds to the proposed *Mesomimivirinae* subfamily within the *Mimiviridae* [58].

**Table 3:** Characteristic features of various *Mimiviridae.*

Name	DNA Pol B	RNA ^2^ Pol II	aaRS ^3^	MCP ^4^	MutS7	AsnS	Transpoviron	Virophage
Mimivirus	Yes	8	4	4	Yes	Yes	Yes	Yes
Megavirus	Yes	8	7	4	Yes	Yes	Yes	Yes
Moumouvirus	Yes	8	5	4	Yes	Yes	Yes	Yes
CroV	Yes	8	1	4	Yes	Yes	No	Yes
PgV	Yes	8	0	2	Yes	Yes	No	Yes
CeV	Yes	8	0	3	Yes	Yes	No	No
TetV	Yes	8	0	1	Yes	No	No	No
BsV	Yes	7	2	4	Yes	Yes	No	No
AaV	Yes	8	0	2	Yes	No	No	No
TupanSL	Yes	8	20	3	Yes	Yes	No	No
TupanDO	Yes	7	20	3	Yes	Yes	No	No
YlmV ^1^	No	D	0	1	No	No	No	No

Abbreviations are the same as in Table 1. ^1^ YlmV: Yellowstone lake mimivirus (NC_028104, 73,689 bp). This metagenomic assembly is erroneously listed as “complete”, while it lacks most of the *Mimiviridae* core genes, including the essential B-type DNA polymerase and most of the transcriptional apparatus. ^2^ Number of distinct encoded RNA polymerase subunits. YlmV only exhibits the minor subunit D. ^3^ Number of encoded amino-acyl tRNA synthetases. ^4^ Number of encoded major capsid protein paralogs.
